# Electronic patient-reported outcome platforms used in hematological cancers: a systematic review

**DOI:** 10.1007/s00520-026-11264-y

**Published:** 2026-09-25

**Authors:** Jessica Nikolovski, Marika Franklin, Eliza A. Hawkes, Louise Acret, Jodie Palmer, Ozgu Serce, Zoe K. McQuilten, Erica M. Wood, Claudia Rutherford

**Affiliations:** 1 Sydney Clinical Trials Centre, University of Sydney, Sydney, Australia; 2 Faculty of Medicine and Health, Susan Wakil School of Nursing and Midwifery, Sydney Quality of Life Office (SQOLO), University of Sydney, Sydney, Australia; 3 School of Public Health and Preventive Medicine, Monash University, Melbourne, VIC Australia; 4 The Daffodil Centre, The University of Sydney, a Joint Venture with Cancer Council NSW, Sydney, Australia; 5 Faculty of Medicine and Health, Susan Wakil School of Nursing and Midwifery, Cancer Care Research Unit, University of Sydney, Sydney, Australia; 6 School of Cancer Medicine, Olivia Newton-John Cancer Research Institute and LaTrobe University, Heidelberg, VIC Australia; 7 Institute for Health and Management, Sydney, Australia; 8 Department of Haematology, Alfred Health, Melbourne, Australia; 9 Department of Haematology, Monash Health, Melbourne, Australia

**Keywords:** Electronic patient-reported outcomes, Hematology, Cancer, Symptom monitoring, Digital health

## Abstract

**Purpose:**

The use of electronic patient-reported outcome measures (ePROMs) has demonstrated benefits in oncology; however, their use in hematological malignancies remains limited. We aimed to identify bespoke ePROM platforms used in hematology settings and describe their features, acceptability, usability, feasibility, implementation and uptake rates, and outcome data.

**Methods:**

A systematic review was conducted following PRISMA 2020 guidelines. Electronic databases (MEDLINE, EMBASE, CINAHL, PsycINFO, Cochrane) and Google Play were searched for studies and mobile applications (apps) reporting on ePROM platforms in hematological cancer populations. Data were extracted about the following: characteristics, features, uses, and patient or clinician-reported data on platform acceptability, usability, feasibility, implementation, and evaluation outcomes.

**Results:**

Fourteen total platforms were identified from 18 studies (reporting 13 unique ePROM platforms) and one platform known to authors. No additional platforms were identified through Google Play search. The included platforms were used in hematological research, clinical care, and registry settings and captured symptoms and quality of life using validated and customized PROMs. Delivery modes included websites and apps that featured automated prompts, clinician alerts, graphical feedback, and electronic medical record integration. Overall, usability was reported to be high; most platforms were acceptable to patients and clinicians. Feasibility and uptake varied, but several studies reported high patient engagement (> 80% completion rates). Positive outcomes included reduced symptom burden, improved treatment adherence, and enhanced patient-clinician communication.

**Conclusions:**

The diversity of identified platforms reflects the wide range of hematological diseases, treatments, and care settings in which ePROMs may be applied, highlighting their flexibility and adaptability. Existing ePROM platforms used in hematological cancers vary substantially in features, purposes, and settings. Future work should focus on tailoring content, supporting implementation, and evaluating the impact of ePROM platforms on patient, clinician, and service outcomes.

**Supplementary information:**

The online version contains supplementary material available at https://doi.org/10.1007/s00520-026-11264-y.

## Introduction

Collectively, hematological malignancies are the second most common group of cancers and their incidence is rising globally, with an increasing number of disease subtypes being identified and treatment paradigms evolving rapidly [1]. Hematological malignancies affect people of all ages and, in 2022, accounted for 6.6% of total cancer cases and 7.2% of cancer-related deaths globally [2]. Within these subtypes, non-Hodgkin lymphoma ranked among the top ten most common cancers, and leukemia had the highest mortality rate [3].

Hematological cancers greatly affect patients’ health-related quality of life (HRQL) due to prolonged and often intensive treatment, symptom burden, and psychological distress [4–6]. Hematology care is increasingly complex and costly with advances in treatment beyond traditional cytotoxic chemotherapies, including molecularly targeted agents, cellular therapies (e.g., CAR T-cell therapy) immunotherapies, and monoclonal antibodies [7]. With this recent proliferation of therapeutic agents, significant different toxicity profiles have emerged, potentially creating unanticipated symptomatic adverse events. Careful monitoring of these is essential, by both patients and clinicians, to make informed joint clinical decisions about treatment choice, supportive care, and dose modification [8].

Patient-reported outcome measures (PROMs) are questionnaires that capture patients’ experiences of symptom burden, treatment side effects, functional status, and HRQL [9]. PROMs are increasingly embedded in routine clinical care, spurred by evidence that they can enhance patient–clinician communication, facilitate earlier identification of adverse events, increase patient satisfaction with care, and correlate with improved clinical outcomes, including survival, HRQL, and reduced emergency visits [10–13]. PROMs are now being integrated into some aspects of routine oncology care to guide individual-level decision-making and in clinical quality registries to support benchmarking and quality assurance activities.

The adoption of internet-capable devices has spurred the transition from paper-based PROM completion to electronic PROMs (ePROMs). Digital collection of PROMs offers numerous advantages: automated data capture, real-time symptom monitoring, improved response accuracy, lower rates of missing data, and potential integration with electronic medical records (EMRs) [14–18]. Several mechanisms underpin the clinical benefit of ePROMs: real-time symptom alerts trigger timely clinical interventions; automated patient feedback fosters self-management; clinicians receive structured data before consultations; and health systems can use aggregated PROM data to support value-based healthcare initiatives [10, 12, 19–21].

Despite growing momentum in solid malignancies, ePROM implementation in clinical practice for hematological malignancies is poorly described [22]. Although many PROMs are being collected electronically using generic survey tools such as REDCap, Qualtrics, or Google Forms for research purposes, these offer limited features and functions that are useful for routine patient-reported outcome (PRO) monitoring (e.g., embedding in medical records, automatic triage, ability for people (e.g., patients, clinicians) outside of the research team to see data). Therefore, this systematic review aimed to synthesize current evidence on ePROM platforms developed specifically for hematological cancer populations. Specifically, it sought to describe their characteristics and features (beyond those offered by existing survey tools for research), the purpose and context of their use, and data on acceptability, usability, feasibility, implementation, and evaluation outcomes.

## Methods

### Study design

This systematic review followed the Preferred Reporting Items for Systematic Reviews and Meta-Analyses (PRISMA) 2020 guidelines (Supplementary File 1). It was not registered nor is there a publicly available protocol.

### Eligibility criteria

Bespoke ePROM platforms were defined as providing functionality beyond questionnaire administration, such as longitudinal symptom tracking, clinician dashboards, alerts, patient feedback, reminders, or electronic record integration. The research question was refined using Sample, Phenomenon of Interest, Design, Evaluation/Outcome, Research type (SPIDER) tool (Table 1), and eligibility criteria were developed based on this. Importantly, the intent of this review was not to characterize ePROM use in research in hematological malignancies, but rather capture bespoke digital platforms used to collect ePROMs routinely. Survey tools such as REDCap or Qualtrics were not included because they offer limited functionality. We did not limit searches or exclude articles based on treatment modality or disease subtypes. Some disease-specific digital health platforms, such as CMyLife, were excluded because they were designed primarily for patient education and medication adherence support rather than routine longitudinal ePROM collection and monitoring for clinical use.

**Table 1:** Inclusion and exclusion criteria used to screen eligibility of identified studies using Sample, Phenomenon of Interest, Design, Evaluation/Outcome, Research type (*SPIDER*) tool

Aspect	Inclusion criteria	Exclusion criteria
**Sample**	• Adult patients diagnosed with any hematological malignancy (e.g., leukemia, lymphoma, myeloma) reporting patient-reported outcomes such as symptoms and HRQL using electronic PROM(s)	• Studies not focused on hematological cancer or hematological cancer patients represented less than 30% of the total sample
**Phenomenon of interest**	• Electronic platforms (including web-based, smartphone applications, and automated voice response systems) used to collect longitudinal PROMs in patients with hematological malignancies	• No PROMs collected • Paper-based PROMs used exclusively • No data about electronic data capture—only clinical outcomes reported • One-off PROM completed • Generic survey tool, rather than bespoke digital platform, used (e.g., REDCap, Qualtrics, Google Forms)
**Design**	• Any study design (randomized controlled trial, prospective cohort studies, qualitative studies, implementation studies, pilot/feasibility studies) or smartphone/digital device application (apps) development studies	• n/a
**Outcome**	• Studies describing acceptability, usability, feasibility, implementation, and/or effectiveness outcome data related to ePROM use	
**Research type**	• Primary, peer-reviewed research published in English • Apps available on Google Play Store in Australia	• Abstracts, reviews, commentaries, or editorials without primary data • Not available in English

*PROMs*, patient-reported outcome measures; *HRQL*, health-related quality of life

#### Electronic search

A comprehensive search was conducted via MEDLINE, EMBASE, CINAHL, PsycINFO, and Cochrane for articles published between 2005 (PRO emergence following USA FDA guidance) and 18 February 2025 (date search performed). Search terms included combinations and different spellings of the following: (“hematologic neoplasm” OR “blood cancer” OR “leukemia” OR “lymphoma” OR “myeloma”) AND (“patient reported outcome” OR “PROM” OR “electronic PROM” OR “ePROM”) AND (“platform” OR “application”). The full search strategy is available in Supplementary Fig. 2. The literature search was supplemented with a search of the Google Play App Store between 12 and 15 August 2025, using hematological cancer terms (Supplementary File 2). Authors identified three platforms through professional knowledge of ongoing hematology ePROM initiatives. These platforms were screened using the same eligibility criteria as those used for publications identified through systematic searching. Only one platform was eligible and included.

### Screening

Retrieved citations were exported into Covidence, a data management software used for systematic reviews (Veritas Health Innovation, Melbourne, Australia). Four reviewers independently screened titles and abstracts (JN, LA, MF, OS). Full-text articles were retrieved for articles labeled “yes” or “maybe” and assessed against eligibility criteria by one reviewer (JN, LA, or MF) with 10% cross-checked by a second reviewer (JN). Discrepancies were resolved by an independent reviewer (CR).

#### Data extraction

A standardized data extraction form was developed to extract study characteristics, platform characteristics and features, and data related to platform acceptability, usability, feasibility, implementation, and effectiveness. Extraction was conducted by JN, MF, LA, and OS. Data were coded as “Yes” only if explicitly described in the publication; otherwise, they were coded as not reported (NR). For the app without a published paper, JN searched the study website and contacted the lead developer, who provided additional information.

#### Risk of bias assessment

Individual study risk of bias was not considered relevant for this review, as we aimed to identify and describe digital platforms used to capture ePROMs rather than to evaluate the quality of the findings in the included studies.

#### Data synthesis

A narrative synthesis was undertaken by JN using guidelines outlined by the Economic and Social Research Council [23]. Microsoft Excel was used to manage data. Information from multiple publications on the same platform was pooled to provide a comprehensive description of the features. When information conflicted between earlier and later iterations, the most recent description was used. Information was collected for any report of a feasibility study or trial that included these data. For trial studies, information was collected on primary and secondary study outcomes and any corresponding results recorded. JN summarized these data and compared findings across identified platforms.

## Results

### Study selection

**Fig. 1:**
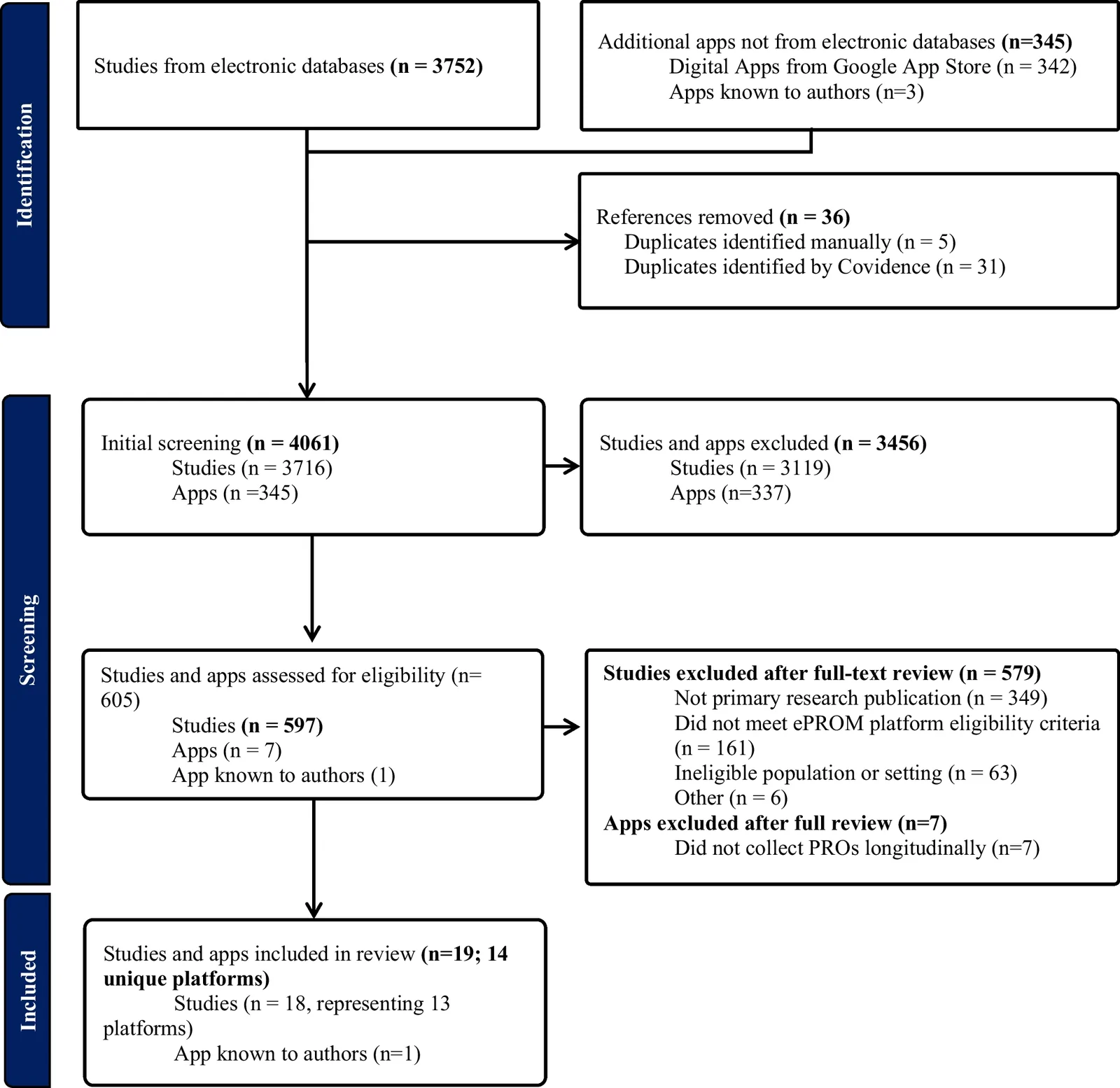
PRISMA flow diagram of selection process for included studies

A total of 3752 records were identified through database searches and 342 through additional sources. After removing duplicates, 4061 records were screened, of which 605 underwent full-text review. Fourteen unique platforms met the inclusion criteria for data extraction. A PRISMA flow diagram (Fig. 1) details the selection process.

### Study characteristics

A total of 18 studies were identified that reported on the use of 13 ePRO platforms in individuals with hematological cancer (see Table 2 for study characteristics) and one additional app was known to the study team that did not have an associated publication at the time of extraction. This meant a total of 14 platforms were synthesized for this review. The studies reporting on the 13 ePRO platforms spanned research (*n* = 9) [24–32], clinical (*n* = 6) [33–38], and registry contexts (*n* = 3) [39–41], and were conducted in eight countries, mainly the USA (*n* = 7) [24, 26, 28–30, 33, 35] and Austria (*n* = 3) [39–41] between 2007 and 2024. Study designs were diverse and included randomized controlled trials, feasibility and pilot studies, prospective observational studies, surveys, qualitative studies, and mixed-methods evaluations.

**Table 2:** Characteristics of ePROM platform

Platform name	Author (year), Country	Use	Study aim	Study design	Health professional population	Patient population	*N* baseline (%blood cancer)	*N* last-time point (% blood cancer)	PRO content	PRO name/validated	Paper completion (%)	Reporting location (home, clinic, anywhere)	Timepoint
Medocity Home Health App	Biran (2020), US	Clinical	Evaluating acceptability and appropriateness of ePRO intervention and explore impact on clinic workflow	Mixed-methods implementation pilot		Relapse-refractory multiple myeloma	11 (100%)	9 (100%)	Physical symptoms	17 items selected from PRO-CTCAE bank (validated)	0	Home	Once a week for 4 weeks
Study web page	Bryant (2020), US	Research	Whether daily use of electronic symptom reporting results automatically fed back to nurses reduced peak symptom burden and improved individual symptoms more than usual care	Quantitative RCT 2-arm (ePRO vs standard care)		HSCT	76 (100%)	72 (100%)	Physical symptoms	PRO-CTCAE (validated)	0	Hospital	Daily for the PRO group. Days + 7, + 10, and + 14 for standard care group
Roskilde Hospital Denmark Haematology registry	Brochmann (2016), Denmark	Research	Investigate where patients were willing and able to use the tool and complete patient-reported outcome measures regularly	Mixed methods feasibility study		MPNs (ET, PV, MF, CML)	118 (100%	104 (100%)	Cancer-specific HRQL, MPN specific symptoms, fatigue	EORTC QLQ-C30, MPN-SAF, BFI, SF-36 (validated)	9	Home	Clinician led
CHES	Efficace (2022), Italy	Clinical	Understand hematologists’ perceptions of usability and clinical utility of this platform in real-life practice	Quantitative—online web survey	23 hematologists	Hematologic malignancies (any) treatment type (any), most CML (*n* = 32, 18%) or MM (*n* = 31, 17%)	189 (100%)	Not applicable	HRQL, symptoms, medication adherence	EORTC QLQ-C30, EORTC Item Library shortened 7-item ARMS-7 (validated)	0	Home	Baseline, every 3 months
CHES	Efficace (2024), Italy, and USA	Research	Evaluate adherence to therapy, assess feasibility, HRQL, patient and physician acceptability, and molecular response	Quantitative international prospective study		ML, aged 18+, newly diagnosed, chronic‐phase CML	93 (100%)	84 (100%)	HRQL, fatigue, social life, satisfaction with care/information	PRO-CTCAE (validated)	0	Clinic	Baseline, every 3 months
MM E-coach	Geerts (2023), Netherlands	Research	Develop and assess application for usability and end-user experiences	Qualitative Action-based development and evaluation pilot study	7 (2 hematologists, 4 nurses, 1 pharmacist)	MM—all treatment including ASCT	20 (100%)	18 (100%)	HRQL and therapy adherence	EORTC QLQ-30, eQ-5D-5L, MARS-5 (validated)	0	Home	3 monthly
Actiwatch Score	Hacker (2007), USA	Research	Describe experiences with EMA and discuss its applicability for capturing real-time, real-world assessments of fatigue in cancer patients receiving intensive therapy	Quantitative Prospective, repeated measures		HSCT	20 before HSCT (100%)	18 after HSCT (100%)	Fatigue	Single-item, global fatigue intensity scale (customized)	0	Home	3 days before and after HSCT: 3 times during the day (6 days total)
Electronic Self-Report Assessment-Cancer (ESRA-C)	King (2013), USA	Research	Describe the prevalence of religious and social struggle and compare to social workers’ assessment	Quantitative—cross-sectional observational study (secondary analysis)		BMT	178 (100%)	178 (100%)	QOL, depression, pain	EORTC QLQ -C30, PHQ-9 ESRA-C (validated)	0	Clinic	NR
Blood Cancer Coach app	LeBlanc (2023), USA	Research	Develop an app designed to help patients with multiple myeloma and chronic lymphocytic leukemia self-manage symptoms and test it for acceptability and preliminary efficacy	Quantitative Pilot RCT		MM or CLL diagnosis	89 (100%)	28 (100%)	Global health, posttraumatic stress, cancer symptoms	10-item PROMIS-29, ESAS-r, PCL-5 (validated)	0	Anywhere	Baseline, 4 weeks, 8 weeks and then emotional distress and symptoms can be reported daily/as needed if patient chooses
CHES	Lehmann (2021), Austria	Clinical/registry	Evaluate patient use of the various components of the portal and aimed to identify patient lack of motivation for not using the patient portal and potential barriers to accessibility	Mixed methods Observational, longitudinal study Semi-structured interviews and counts of portal pages accessed		MM or CML, outpatient treatment	102 (100%)	71 (100%)	Symptoms, functional health, global QOL	EORTC QLQ-C30 MY20, or CLL17 (validated)	0	Anywhere	The week before hospital visits (regular, ranging 1 week to 12 months) Patients encouraged to complete questionnaires as often as they like, even daily
CHES	Lehmann (2023), Austria	Clinical/registry	Investigate adherence to and patient perceptions of a weekly, web-based patient-reported outcome measure symptom monitoring program in routine clinical practice and how clinical alerts prompted by the system influenced clinical care	Quantitative Longitudinal observational		MM	39 (100%)	35 (100%)	Symptoms most relevant to MM, core symptom set (pain, fatigue, nausea, vomiting, constipation, diarrhea, polyneuropathy, emotional symptom burden, sleep disturbances, global health) + treatment-specific (e.g., rash, edema, fever)	EORTC QLQ-30 and EORTC QLQ-MY20 (validated)	0	Anywhere	Weekly EORTC QLQ-30, EORTC QLQ-MY20 at baseline and every 6 weeks. Patients can also report anytime
ePRO HESA platform	Nikitina (2024), Russia	Clinical	Evaluate the feasibility of incorporating ePROs into routine clinical practice through HESA	Quantitative feasibility—exploratory study		LN receiving aHSCT	95 (100%)	95 (100%)	QOL impact, signs and symptoms, depression, and anxiety	HM-PRO, HADS (validated)	0	Anywhere	Before aHSCT, at discharge, after discharge
Carevive PROmpt™	Patel (2024), USA and Canada	Clinical	Longitudinally characterise the MM treatment experience in detail based on line of therapy	Quantitative prospective, observational, multi-site longitudinal study		MM—any stage and anywhere within the treatment continuum	223 (100%)	153 (100%)	Symptom burden, HRQL	FACT-GP5, EORTC QLQ-C30 Item 30 (validated)	0	Anywhere	Weekly
My Hospital App	Rosenberg (2024), Denmark	Clinical research	Develop a questionnaire addressing side effects and an algorithm stratifying patients according to their responses and test the usability of electronic PRO data to form the basis of decision-making on whether patients are physically fit for the next treatment with an unchanged dose and if some patients might need additional support from healthcare professionals	Mixed method Prospective Quantitative—patient PRO data, Qualitative —individual interviews with patients, focus group with healthcare professionals	3 nurses and 2 physicians	People with M-component dyscrasias scheduled for bortezomib treatment	44	33	Common side effects of bortezomib; neuropathy, fever, diarrhea, constipation, and other side effects interfering with treatment	Customized	0	Home	NR
ReLive	Song (2021), Korea	Clinical	Usability test to assess the ease of use and understanding, usefulness, and acceptability of the ReLive application among survivors with CML before conducting a study on its effectiveness	Mixed-methods Explorative descriptive study design	3 nurses	CML, molecular remission Males only	10 (70%)	10 (70%)	Social support, self-efficacy, and QOL	NR	0	Anywhere	NR
INternet-based Survivorship Program with Information and Resources (INSPIRE)	Syrjala (2018), USA and Canada	Research	Examine the efficacy of INSPIRE, an INternet-based Survivorship Program with Information and REsources, with or without problem-solving treatment (PST) telehealth calls, for survivors after hematopoietic cell transplantation (HCT)	Quantitative RCT		MM	755	651	Cancer and treatment distress, depressive symptoms, physical dysfunction, fatigue	CTXD, SCL-90-R, SF-36, FSI (validated)	0	Home	NR
CHES	Sztankay (2019), Austria	Registry	Depict the implementation procedure and to evaluate the feasibility and usability of routine ePRO monitoring for integrating patient‐reported QOL data with data of the Austrian Myeloma Registry (AMR) at a single participating center. Additionally, differences in attitudes towards routine ePRO assessment between new and more experienced users are evaluated	Mixed method evaluation and feasibility study		MM	140 (100%)	127 (100%)	Physical, role, emotional, social and cognitive, QOL, disease symptoms, treatment side effects, functioning—future perspective, body image, mobility, self‐care, usual activities, pain/discomfort, and depression/anxiety	EORTC-QOL – MM, EORTC QLQ‐MY20, EQ‐5D‐5L (validated)	0	NR	About half pts completed 2–5 (up to 22 times)
Patient-reported outcome quality of life (PROQOL) tool	Warsame (2022), USA	Clinical	Determine if the PROQOL tool improved patient QOL over usual care at the end of 12-month follow-up	Quantitative Single-center, non-blinded prospective clinical trial; randomized patients (2:1)		MM light chain AL, head and neck cancer, gynecologic malignancies	383 (61%)	338 (66%)	Personal relationship, emotional health, physical health, cancer diagnosis and treatment, health behaviors, money, care-planning	PROQOL (customized) LASA scales for QOL of various domains (validated)	0	Clinic	Before every visit Maximum 1/week
	No paper—Hodgkin MyHealth (MBILE APPcollect vital data on lymphoma treatment, remission status, and medical issues including fertility for patients living with Hodgkin lymphoma), Australia	Research	Not applicable	Feasibility trial		Australian Hodgkin lymphoma current patients and survivors	246 (100%)	246 (100%)	Lymphoma status, past treatment(s), toxicities (cardiac, respiratory, neurological, hormonal, fertility, secondary malignancies)	Customized	0	Anywhere	Baseline, shorter updates 6-monthly

*AL*, amyloidosis; *aHSCT*, autologous hematopoietic stem cell transplantation; *ARMS-7*, Adherence to Refills and Medications Scale; *ASCT*, autologous stem cell transplantation; *BFI*, Brief Fatigue Inventory; *BMT*, bone marrow transplantation; *CLL*, chronic lymphocytic leukemia; *CLL17*, chronic lymphocytic leukemia with a 17p deletion; *CML*, chronic myeloid leukemia; *CTXD*, cancer and treatment distress; *EORTC QLQ-C30*, European Organisation for Research and Treatment of Cancer Quality of Life Questionnaire –30; EORTC QLQ-MY20, European Organisation for Research and Treatment of Cancer Quality of Life Questionnaire – Multiple Myeloma; *EQ-5D-5L*, EuroQol-5 Dimension 5-level; *ePROs*, electronic patient-reported outcomes; *ESAS-r*, Edmonton Symptom Assessment System – Revised; *ESRA-C*, Electronic Self-Report Assessment—Cancer; *ET*, essential thrombocythemia; *FACT-GP5*, Functional Assessment of Cancer Therapy – General item GP5; *FSI*, Fatigue Symptom Inventory; *HADS*, Hospital Anxiety and Depression Scale; *HESA*, health electronic self-assessment; *HM-PROM*, hematological malignancy patient-reported outcome measure; *HRQL*, health-related quality of life; *HSCT*, hematopoietic stem cell transplantation; *LASA*, linear analogue self-assessment scales; *LN*, lymphoproliferative neoplasms; *MARS-5*, Medication Adherence Report Scale – 5 item version; *MF*, myelofibrosis; *ML*, myeloid leukemia; *MM*, multiple myeloma; *MPN-SAF*, Myeloproliferative Neoplasm Symptom Assessment Form; *MPL*, myeloproliferative neoplasms; *NR*, not reported; *PCL-5*, Posttraumatic Stress Disorder Checklist for DSM-5; *PRO*, patient-reported outcome; *PRO-CTCAE*, Patient-Reported Outcomes version of the Common Terminology Criteria for Adverse Events; *PROQOL*, Patient Reported Outcome Quality of Life; *PROMIS-29*, Patient-Reported Outcomes Measurement Information System-29; *PV*, polycythemia vera; *QOL*, quality of life; *RCT*, randomized control trial; *SCL-90-R*, Symptom Checklist-90-R depression scale; *SF-36*, Short Form 36 Health Survey

Studies reported diverse hematological cancer populations, most commonly among patients with multiple myeloma (MM), with sample sizes ranging from 10 to 755. Chronic myeloid leukemia (CML) was also frequently studied, either exclusively or within mixed cohorts (*n* = 118 [25]; *n* = 189 [37]; *n* = 93 [26]; *n* = 10 [34]; *n* = 102 [39]). Other hematological malignancies included chronic lymphocytic leukemia (non-Hodgkin lymphoma *n* = 89) [30], and myeloproliferative neoplasms such as essential thrombocythemia, polycythemia vera, and myelofibrosis (*n* = 118 [25]), lymphoproliferative neoplasms (*n* = 95 [36]), and complications of malignancies including light chain amyloidosis (*n* = 383 [33]). Several studies focused on patients undergoing hematopoietic stem cell transplantation (HSCT) or bone marrow transplantation, with sample sizes ranging from 18 to 178 (*n* = 76 [24]; *n* = 20 [28]; *n* = 178 [29]; *n* = 755 [32]), and individuals with M-component dyscrasias receiving bortezomib (*n* = 44 [31]).

A wide range of PRO domains were captured, including physical symptoms, HRQL, fatigue, emotional distress, and treatment adherence. Validated PROMs such as the European Organisation for Research and Treatment of Cancer Quality of Life Questionnaire Core 30 (EORTC QLQ-C30), Patient-Reported Outcomes version of the Common Terminology Criteria for Adverse Events (PRO-CTCAE), Patient-Reported Outcomes Measurement Information System 29-item profile (PROMIS-29), and Hematological Malignancy Patient-Reported Outcome Measure (HM-PRO) were commonly used, though some studies employed custom-designed PROMs (*n* = 6). Reporting locations were either at the clinic or home, with most studies allowing flexible completion based on patient preference. Four studies reported on the frequency of ePROM completion, ranging from daily to every 3 months; some studies offered patient- or clinician-led timepoints and frequencies of PROM completion (*n* = 4) [25, 30, 39, 40].

## ePROM platform features

Platform features are summarized in Table 3. The 14 ePROM platforms included commercial systems (e.g., The Computer-based Health Evaluation System (CHES) used in five studies in clinical and registry settings) [26], and custom-built interfaces. Platforms were primarily delivered via web portals and apps for smartphones and tablets. Integration with clinical workflows varied; only two platforms, both used in registries, were embedded within EMRs [29,. The others operated as standalone platforms not integrated in EMRs or registries.

**Table 3:** PRO digital technology/platform features for published studies

Platform name	Paper(s) reporting platform	Prompt for Pt	Reminders to Pt	Result reports for HP	HP remote access	Pt see results?	HP alerts	Tailored Pt advice	General Pt advice	Pt–pt communication	Automatic referral?	EMR integrated
Study web page	Bryant (2020)	NR	NR	Y- scores	Y	NR	NR	NR	NR	NR	NR	NR
Roskilde Hospital Denmark Haematology registry	Brochmann (2016)	Y	Y	Y- graph	NR	N	NR	NR	NR	NR	NR	Only integrated with blood test results
Medocity Home Health App	Biran (2020)	Y	NR	Y- graph and score 1–4	Y	Y	Y	Y	NR	NR	NR	N
CHES	Efficace (2022)	Y	Y	Y- colored bar graph	Y	Y	Y	NR	NR	NR	NR	NR
	Efficace (2024)	NR	NR	Y- colored bar graph	NR	NR	NR	NR	NR	NR	NR	NR
	Lehmann (2021)	NR	NR	Y- colored bar graph	NR	Y	NR	NR	Y	NR	NR	NR
	Lehmann (2023)	NR	Y	Y- colored bar graph	NR	Y	Y	Y	Y	NR	NR	NR
	Sztankay (2019)	NR	NR	Y- graph with comparison values from local and reference cohorts	NR	NR	NR	NR	NR	NR	NR	NR
MM E-coach	Geerts (2023)	NR	NR	Y- “red flag”	Y	NR	Y	Y	Y	Y	N	NR
Actiwatch Score	Hacker (2007)	Y	NR	NR	NR	NR	NR	NR	NR	N	N	NR
Electronic Self-Report Assessment—Cancer (ESRA-C)	King (2013)	NR	NR	Y- scores (0–10 scale), bar graph for religious/social struggle	Y	NR	NR	NR	NR	NR	NR	Y
Blood Cancer Coach mobile app	LeBlanc (2023)	Y	NR	NR	NR	Y- graph	NR	Y	Y	NR	NR	NT
ePRO HESA platform	Nikitina (2024)	Y	NR	NR	NR	Y	NR	Y	Y	NR	NR	NR
Carevive PROmpt™	Patel (2024)	NR	NR	NR	NR	NR	Study team notified oncology team of severe or very severe scores	Y	NR	NR	NR	NR
My Hospital app	Rosenberg (2024)	NR	NR	Y- traffic light system	NR	NR	Concerns appeared red	NR	NR	NR	NR	Y
ReLive	Song (2021)	NR	Y	Y	NR	Y	NR	NR	NR	NR	NR	NR
INternet-based Survivorship Program with Information and Resources (INSPIRE)	Syjala (2018)	NR	NR	NR	NR	NR	NR	Y	Y	Y	NR	NR
Patient-reported outcome quality of life (PROQOL) tool	Warsame (2023)	NR	NR	NR	NR	NR	NR	Y	N	N	N	N

Clinician alerts were defined as notifications or flagged scores intended to draw clinicians' attention to concerning patient-reported symptoms, whereas automatic referrals were defined as the automated routing of patients to supportive care services without clinician review

*NR*, not reported; *Y*, yes; *N*, no

Six platforms (reported in five studies and one app), used in registry and clinical settings, included automated prompts (email, SMS, or push notifications) to notify patients to complete PROMs when they were due [25, 28, 36–38]. For example, in clinical settings, the ePRO Health – Electronic Self-Assessment (HESA) platform sent an email [36] and the Medocity Home Health App offered email, text, or push notifications based on patient preference on the day PROMs were scheduled [38]. Additionally, three platforms (CHES, registry and clinical setting, Roskilde Hospital Denmark Hematology registry; ReLive, clinical setting; and Hodgkin MyHealth, research) described the use of reminder systems if PROMs were not completed by the due date, with SMS, email, or push notification reminders typically sent after 1 day or 1 week [25, 34, 37, 40].

Eight platforms described how PRO data were presented, including to patients (*n* = 5: Medocity Home Health App, CHES, ePRO HESA platform, ReLive, MyHodgkin MyHealth) [34, 36–40] and clinicians (*n* = 4: all studies except Actiwatch Score, ePRO HESA platform, Carevive PROmpt™, INSPIRE, PROQOL tool) [24–27, 29, 31, 34, 37–41]. Formats included bar graphs or trend lines (*n* = 8), raw scores (*n* = 3), traffic light color coding (*n* = 2), and a text summary of responses to patients (*n* = 1). For instance, the research study web page by Bryant et al. presented scores [24], and My Hospital App used a traffic light system to flag concerning scores [31], while Electronic Self-Report Assessment-Cancer (ESRA-C) provided a combination of scores on a 0–10 scale and a bar graph for religious and social struggle [29]. Six studies, all in clinical settings, reported that patients tracked their PROM data typically through graphs [34, 36–40]. Five platforms allowed health professionals to remotely access and monitor PROs, through secure web portals in research and clinical settings or EMR integration in registry settings [24, 27, 29, 37, 38].

Four platforms, across clinical and research settings, included features to alert clinicians when patients reported severe or worsening symptoms (Medocity Home Health App, CHES, MM E-coach, My Hospital App) [27, 31, 37, 38, 40]. Efficace et al. (2022) reported that their platform’s alerts were manually actioned by clinicians in the form of an ad hoc hospital appointment or video consultation and/or a referral to supportive care, with feedback back to patients if needed [37]. Carevive PROmpt™, used in a research study, described a process whereby study researchers notified oncology teams of “severe” or “very severe” scores, so patients received standard care to address their symptom burden [35]. No study reported whether PROs were aggregated at a clinic or service level.

Seven platforms in clinical and research settings provided tailored advice to patients based on their responses (e.g., self-management tips for fatigue or nausea) [27,. In contrast, four platforms in clinical and registry settings offered general educational content about cancer and treatment-related symptoms, without personalization [27, 32, 36, 39, 40].

Two platforms, both used for research, included a peer support forum for patients to communicate with one another [27, 32].

No platforms reported automatic referral mechanisms, where concerning or worsening symptoms triggered referrals to supportive care services. Two platforms, both used in research, described integration with EMRs so clinicians could view symptom scores alongside other clinical information [29, 31]. 

## Acceptability, usability, feasibility, implementation, and clinical outcomes

Across the included studies, platform acceptability, usability, and feasibility were frequently reported, with several also examining downstream impact on patient HRQL outcomes. Common themes reported in studies included high patient satisfaction, ease of use, and clinician endorsement.

### Acceptability

Using digital PROs across clinical, research, and registry settings was generally reported as acceptable. Patients with myeloproliferative neoplasms found the number of PROM questions acceptable in the platform used by the Roskilde Hospital Denmark Hematology registry [25]. Similarly, patients reported that the frequency, time taken, and training offered to complete PROMs within the Medocity Home Health App were acceptable. While 75% of patients with multiple myeloma were “not at all” and 25% “a little” burdened by completing PROMs [40], there was reduced motivation to complete PROMs in future when results were not addressed during consultations by their clinician [39]. Ninety-seven percent of patients with M-component dyscrasias scheduled for bortezomib treatment recommended the app with 100% wishing to continue registering their side effects in the app post study [31]. Similarly, 84% of patients with multiple myeloma were willing to use the CHES, but only 37% continued to use the portal across all three planned study timepoints [39]. In another study using the CHES, the platform was reported to be acceptable for patients and clinicians alike [26]. No difference was found in acceptability (*p* = 0.619) between Internet users and non-users completing PROMs in a registry setting, with 87.5% patients with multiple myeloma reporting being “quite” to “very” satisfied with the ePRO monitoring procedure [41]. Additionally, 80% of clinicians indicated being “quite” to “very satisfied” with ePRO monitoring in the registry setting [41].

#### Usability and technical functionality

Usability was generally rated positively, though some limitations to platform functionality were noted. Patients reporting symptoms using Biran et al.’s study webpage understood weekly reporting, but were confused by the feature to report symptoms ad hoc, prompting clinicians to suggest the removal of this option in favor of fixed timepoints for completion [38]. In one study, patients with multiple myeloma scored the MM E-coach platform 60/100 on usability, with feedback focused on improving flexibility and design [27]. In a registry, most patients with multiple myeloma had no issues with tablet use, although patients categorized as internet non-users required more assistance than internet users [41]. Another study similarly reported older male patients with chronic myeloid leukemia and molecular remission were less comfortable with smartphones, but all found the app easy to use once familiar [34]. No technical issues were reported with the Roskilde Hospital Denmark Hematology registry platform, though one patient required assistance due to visual impairment [25]. Efficace et al. (2022) reported all hematologists agreed the CHES platform was easy to use for care of patients with myeloid leukemia with newly diagnosed chronic myeloid leukemia and 78% of hematologists accessed the CHES platform monthly to review data [37]. Rosenberg et al. reported that nurses were initially unfamiliar with using digital platforms for PRO collection, but usability improved over time as nurses became more familiar with how the platform worked [31].

#### Feasibility and workflow integration

Feasibility and workflow varied across studies and settings. Efficace et al. (2022) reported, across Italian academic medical and clinical trial centers for blood diseases, there were many different IT structures, but the CHES was successfully integrated in routine care [37]. However, in another study also using the CHES, they did not meet a feasibility benchmark which was 80% questionnaire completion at > 60% of visits [26]. This was largely attributed to COVID-19 disruptions in a population of patients with any hematological malignancy [26]. Another study examining ePROM collection for patients receiving HSCT demonstrated strong feasibility for real-time fatigue assessments, maintaining high response rates even among acutely ill patients [28]. There were also logistical considerations reported in one registry setting such as device disinfection and room availability, though these minimally delayed clinical routines [41].

Time and effort required for platform use were reported as generally manageable. Five studies reported the time taken for patients to complete PROMs, which included averages of 9.4 minutes per portal session [39], under 6 minutes [34] and 2.9 minutes [33]. Clinicians took up to 30 minutes to set up and instruct each patient as a one-off and 3.2 minutes to review their patients’ PROMs [31]. Lehmann et al. adjusted monitoring intervals for patients who found weekly assessments burdensome [40]. Rosenberg et al. reported that the My Health App led to perceived reductions in workload and that nurses reported high confidence in the algorithm to score symptoms and alert them of concerning symptoms, using a traffic light system, for timely care [31].

#### Implementation and uptake

Implementation success and uptake rates were inconsistently reported. PRO completion rates varied 70.3% [40] to 97% (84% of patients completing the PROs at least twice post-HSCT) [36]. Patients treated with HSCT accessing the Actiwatch Score app maintained high engagement even during acute illness phases [28]. Bryant et al. reported 10 dropouts over 6 months [26] due to death, Lehmann et al. (2021) reported lower rates of completion over time in an outpatient treatment center due to missed appointments and lack of automated reminders (added to the platform post-study) [39].

### Patient outcomes

Some studies reported downstream outcomes, but heterogeneity in outcome reporting limited direct comparison. Positive outcomes associated with ePROM use were reported where patients receiving the PRO intervention experienced a significantly lower symptom burden following HSCT (10.4 vs. 14.5 symptoms, *p* = 0.03) [24] and high medication adherence with ePRO use, with 92.5% of myeloid leukemia patients taking ≥ 90% of prescribed doses [26]. Similarly, HRQL improved or stabilized for 69% of HSCT patients post-discharge [36] and distress improved in the intervention arm where multiple myeloma patients received telehealth calls to manage issues related to their treatment triggered by their PROM scores (*p* = 0.032), with further improvements from higher engagement in the platform and symptom reporting [32].

Patient perceptions of patient-clinician communication were reported, where 78% of patients with multiple myeloma felt their care team was better informed, and 55% appreciated follow-up calls [40], mirroring Sztankay et al. who also highlighted high satisfaction and perceived value from multiple myeloma patients in informing clinicians of their health status [41]. Warsame et al. (mixed cancers, including multiple myeloma and light chain amyloidosis haematologic subtypes) reported increased diversity in patient concerns over time (i.e., more symptoms reported with each assessment) (*p* = 0.001) [33].

## Discussion

This systematic review identified 14 distinct ePROM platforms used in hematological cancer care across clinical, research, and registry settings. Many studies were excluded from this review because they relied on generic survey tools such as REDCap, Qualtrics, or Google Forms, which primarily support data collection for research purposes and offer limited functionality for clinical use. In contrast, this review focused on bespoke digital platforms that provide enhanced capabilities, such as longitudinal tracking, real-time visualization, and alerting, enabling clinicians and patients to monitor PROs over time and integrate these insights into routine care. Reviewed platforms varied by which populations they were developed for and used with, delivery mode, integration with clinical workflows, and features such as symptom alerts, graphical visualization of data, and tailored advice. Where relevant to their study’s aim, usability, acceptability, and feasibility were detailed for some platforms. Overall, patients and clinicians reported that the platforms were user-friendly and acceptable for use in clinical care settings, and were feasible within existing workflows. Synthesizing available apps is an important step toward implementing ePROs into hematological cancer care, as it leverages the substantial time and institutional resources already invested, including secure portal development, content creation, pilot testing, and establishing new security and ethical standards [42].

Existing qualitative and implementation studies on cancer-related apps report that patients generally value digital platforms that support self-management, symptom tracking, and communication with clinicians, with intuitive interfaces and personally relevant symptom questions [43, 44]. However, patients with cancer report that unwanted notifications, reminders of illness, and a lack of human interaction can reduce the acceptability of mobile apps [43]. This is mirrored in other contexts, such as ambulatory surgery, where one of the top necessary features for patients was communication with healthcare professionals [45]. Similarly, patients with rheumatoid arthritis preferred platform features that tracked symptoms, scheduled appointments, and provided lab results; in contrast, social support features (e.g., peer forums) were rated less useful [44]. Cancer patients have previously reported unmet information needs during consultations, prompting suggestions for features like question prompt lists, glossaries, and resource directories to improve understanding of their cancer and reduce anxiety associated with clinical appointments and what to expect in the future [46]. While cancer patients value tailored feedback on managing their symptoms, only a subset of the hematological ePROM platforms provide personalized advice; others offer generic educational content, which may be less useful for self-management.

Clinicians, meanwhile, emphasize the importance of ePROM platform integration with electronic medical records, real-time alerts for severe or worsening symptoms, and structured data presentation to support clinical decision-making [47]. However, our study reported only two platforms embedded within EMRs, which limited seamless integration into routine workflows. Most operated as standalone platforms, requiring separate logins or manual data transfer, which may hinder clinician uptake in hematological settings. Many platforms included in this review offered automated prompts for completion and graphical displays of data that help provide timely, actionable information to inform care. A notable gap, however, is the absence of automatic referral mechanisms. Although the literature highlights the potential of ePROMs to facilitate timely access to supportive care [15], none of the platforms in this review included features that triggered referrals based on symptom severity. This omission may reflect technical or organizational barriers and remains an issue to be addressed to enhance care coordination and responsiveness.

A large body of literature indicates that PROMs are considered valuable for conveying patient experiences to clinicians. We found heterogeneity in which PROMs were collected in hematological contexts; both generic and condition-specific PROMs were used, alongside some customized questionnaires. In the management of chronic conditions more broadly, patients and clinicians tend to prefer disease-specific rather than generic PROMs because generic PROMs are perceived to inadequately capture the lived experience of rare or complex conditions [47–49], which includes hematological cancers. Further, generic measures may be less sensitive to specific disease and treatment effects. Research is needed to identify which PROMs capture the PROs most valuable to patients and clinicians in a hematology context, considering new and emerging treatments. Determining which PROM(s) are best suited to the needs of hematological patients, and standardizing across settings, can facilitate clinical decision-making at an individual level, and enable aggregated data for quality assurance and benchmarking activities at a service and policy level.

### Strengths and limitations of this study

A major strength is the comprehensive search strategy, including peer-reviewed literature and app store searches. We limited searches to English publications and published descriptions of platform features. Our review did not comprehensively search for evaluation and implementation outcomes data published outside peer-reviewed or in gray literature; usability and implementation findings were extracted only when explicitly reported in the included studies. Other sources, including app websites and conference abstracts, were not searched owing to resource constraints and the review’s focus on peer-reviewed evidence. It is possible that ePROM platforms are being used in clinical settings without published reports or papers. As methodological quality and risk of bias were not assessed, findings relating to acceptability, feasibility, usability, and clinical outcomes should be interpreted cautiously. Therefore, while we present valuable insights into platform functionality and user experience, these findings should be interpreted as indicative rather than exhaustive.

### Limitations of the available evidence

There is a lack of data on sustained use, long-term implementation, and their associated costs. Few studies reported whether platforms were used beyond the study period or how they were maintained in clinical or registry settings. This limits our understanding of the scalability and sustainability of ePROM platforms in hematology. Future research should prioritize longitudinal evaluations of platform uptake, integration, and impact on clinical workflows and patient outcomes. Furthermore, there was limited focus on the use of platforms by culturally and linguistically diverse populations, which is an identified gap in PROM implementation globally [50]. Several platforms incorporated customized questionnaires; however, reporting of questionnaire development was limited, preventing consistent determination of whether these were adapted from existing validated PROMs, derived from item banks, or newly developed for platform-specific use. Finally, there is little empirical evidence to inform optimal and preferred features of ePROM platforms for use in future hematological cancer research, registries, or practice.

#### Future directions

Future directions for integrating ePROMs in hematological cancer care include tailoring and validating content to capture hematology-specific symptoms and treatment effects; optimizing the timing of PROM collection to align with clinical trajectories and individual patient preferences; and, rigorously evaluating the impact of ePROM use on clinical outcomes, patient experience, and health service delivery. Embedding PROMs into registries and real-world data systems will support benchmarking and quality improvement, and ensure patient voices are meaningfully integrated into care and research, as reported in emerging initiatives in Australia [51]. This study was limited to adults; future work warrants similar reviews in platforms suitable for caregivers and pediatric and child patient populations, who may have different comfort levels with reporting PROs and using technology [52]. Our future work, guided by this study, will provide practical suggestions and approaches for ePROM platform integration in hematological cancer care.

Our findings highlight the importance of tailoring ePRO platform design to meet the diverse needs of patient populations, including those with visual impairments or limited confidence in technology. Considerations for app developers may include features like adjustable font sizes, high-contrast interfaces, voice-assisted navigation, and simplified layouts. Moreover, building user confidence through onboarding tutorials, intuitive workflows, and responsive technical support can enhance usability and acceptability.

## Conclusion

By centering the patient voice through PROs, ePROM platforms are a promising resource for hematological cancer care. The diversity of platforms identified demonstrates their potential application across research, clinical care, and registry settings. Further evaluation is needed to determine how best to implement and sustain these platforms in routine care.

## Supplementary information

Below is the link to the electronic supplementary material.

ESM 1 (PDF 119 KB)

ESM 2 (DOCX 15.2 KB)

## Data Availability

No datasets were generated or analysed during the current study.
