# Supplementary material for: Electronic patient-reported outcome platforms used in hematological cancers: a systematic review
Source: Support Care Cancer. 2026 Sep 25;34(10):1018. doi: 10.1007/s00520-026-11264-y (PMC13615055; doi:10.1007/s00520-026-11264-y)
Supplement: Supplementary file 2 [file 520_2026_11264_MOESM2_ESM.docx]

**SEARCH STRATEGY**

**Haematological cancer**

1. hematologic malignancy/exp
2. hematologic malignanc*.kw,ti,ab
3. haematologic malignanc*.kw,ti,ab
4. hematological malignanc*.kw,ti,ab
5. haematological malignanc*.kw,ti,ab
6. hematological cancer*.kw,ti,ab
7. haematological cancer*.kw,ti,ab
8. hematologic neoplasms.kw,ti,ab
9. haematologic neoplasms.kw,ti,ab
10. blood cancer*.kw,ti,ab
11. leukaemia.kw,ti,ab
12. lymphoma.kw,ti,ab
13. myeloma.kw,ti,ab
14. myelodysplastic syndrome*.kw,ti,ab
15. myeloproliferative neoplasm*
16. waldenstrom* macroglobulinaemia.kw,ti,ab
17. histiocytosis.kw,ti,ab
18. erdhein*chester disease.kw,ti,ab
19. mastocytosis.kw,ti,ab
20. or/1-19

**Digital systems**

21. medical Informatics.kw,ti,ab
22. telemedicine.kw,ti,ab
23. applications.kw,ti,ab
24. Smartphone.kw,ti,ab
25. electronic.kw,ti,ab
26. online.kw,ti,ab
27. web-based.kw,ti,ab
28. remote or remotely.kw,ti,ab
29. monitor or monitoring.kw,ti,ab
30. screen or screening.kw,ti,ab
31. or/21-30
32. Patient report* or Patient-report* or Self report* or Self-report* or Self manage* or Self-manage* or Self monitor* or Self-monitor* or self assess* or self-assess* or symptom.mp.
33. 31 and 32
34. Limit 33 to (humans and yr=“2005 -Current”)
